# Complete genome sequence of *Saccharolobus solfataricus* strain S441, from Lassen Volcanic National Park

**DOI:** 10.1128/mra.00288-25

**Published:** 2025-06-23

**Authors:** Jonathan Abshier, Caleb Marceau, Kenneth M. Stedman

**Affiliations:** 1 Department of Biology, Portland State University 170365, Portland, Oregon, USA; 2 Center for Life in Extreme Environments, Portland State University 6685, Portland, Oregon, USA; University of Southern California, Los Angeles, California, USA

**Keywords:** archaea, extremophiles, viruses

## Abstract

Here, we present the complete genome sequence of *Saccharolobus solfataricus* strain S441, isolated from Devil’s Kitchen, Lassen Volcanic National Park in Northern California, U.S.A. The genome for this strain is 2,766,550 base pairs, with a GC content of 35.7% and 3,031 genes.

## ANNOUNCEMENT

Hyperthermophilic archaea of the genus *Sulfolobus/Saccharolobus* thrive in acidic, high-temperature environments such as terrestrial hot springs and volcanic fields (1). These extremophiles serve as valuable model organisms for studying archaeal cell biology, DNA replication, and genome evolution under extreme conditions (2). Here, we describe the complete genome of *Saccharolobus solfataricus*, strain S441 (CP160827.2), isolated from a volcanic hot spring in Devil’s Kitchen, Lassen National Volcanic Park, Northern California. Strain S441 has been used extensively as a host for fuselloviruses (3)

Strain S441 was isolated from a sample collected from an 83.6°C, pH 2 hot spring in Devil’s Kitchen, Lassen Volcanic National Park on 4 July 2008 (see Table 1 for GPS coordinates). The sample was adjusted to pH 5 with solid calcium carbonate on site and transported anaerobically to Portland, OR, at ca. 25°C. The sample was enriched for 5 days aerobically in Sulfolobus medium (4) at 80°C. Strain S441 was isolated as a single colony by plating the enrichment culture on 1% Gelrite plates aerobically. Stocks were stored in Sulfolobus medium with 50% glycerol at −80°C. For DNA preparation, S441 was grown from stocks in 1× DT media (5) (pH: 3.5) at 80°C in a dry incubator for 5 days to an OD600nm of 0.3. Cells were pelleted in a Thermo Sorvall Lynx 6000 floor centrifuge at 20,217 RCF for 5 minutes, and chromosomal DNA was extracted using a modified protocol (6). Briefly, cell pellets were resuspended in TEN (10 mM Tris-HCl pH 8.0, 1 mM EDTA, and 150 mM NaCl) followed by the addition of an equal volume of TEN with 1.6% sarcosine and 0.12% Triton X-100. After a 30-minute incubation at room temperature, genomic DNA was purified via phenol/chloroform/isoamyl alcohol extraction, followed by ethanol precipitation. The resulting DNA pellet was washed with 70% ethanol, air-dried, and resuspended in nuclease-free water. DNA integrity was confirmed by nanodrop spectrophotometry, digestion with EcoRI, and analysis via agarose gel electrophoresis.

**TABLE 1:** Sample location, annotation, and genome links for *S. solfataricus* S441

Strain characteristic(s)	*S. solfataricus*, S441
Sampling site	Devil’s Kitchen, Lassen Volcanic National Park
Sampling coordinates	40.4333 N 121.6936 W
Genes (total)	3,031
CDSs (total)	2,979
rRNAs	5S, 16S, and 23S
tRNAs	47
ncRNAs	2
CRISPR Arrays	5
No. of contigs	1
Genome length (bp)	2,766,550
Genome coverage	39.0×
BioSample ID	SAMN42077069
BioProject ID	PRJNA1128679
GenBank ID	CP160827.2
Raw Reads	SRX27502923

Purified chromosomal DNA from strain S441 was sequenced by Eurofins Genomics. Library preparation used Oxford Nanopore Technologies (ONTs) Rapid Barcoding Kit V14, and sequencing utilized an ONT R10.4.1 flow cell on a GridION instrument, running MinKnow. Base calling was performed using Dorado v0.7.4 (ONT). No size selection was performed prior to sequencing, and post-sequencing read filtering was conducted using SeqKit (7) to remove low-quality reads (<Q7), and then subsampled from the remaining high-quality reads. A total of 78,631 raw reads (N50 = 6,172 bases) were generated and assembled using Flye v2.9.55 to produce and circularize the final genome sequence (8). Genome circularity was confirmed with Bandage v0.9.0 (9). The genome was not rotated. For sequencing and annotation details, see Table 1. The assembled genome was annotated using the NCBI Prokaryotic Annotation Pipeline, which identified coding sequences, rRNAs, tRNAs, and other genomic features (10). Pairwise average nucleotide identity (ANI) analysis was conducted using FastANI (11) to assess the genomic relatedness of *S. solfataricus* strain S441 to other publicly available *Saccharolobus* genomes. Default parameters were used for all software. Among the genomes analyzed, *S. solfataricus* strain P1 (LT549890.1) exhibited the highest ANI value (98.63%) with strain S441, indicating that P1 is its closest known relative based on whole-genome similarity. This genomic resource expands the available sequence data for *Sulfolobus/Saccharolobus* species and supports future research into archaeal genetics, virology, evolutionary biology, and adaptations to extreme environments.

## Data Availability

The complete genome sequence of *Saccharolobus solfataricus* strain S441 has been deposited in GenBank under the accession number CP160827.2. Associated BioProject BioSample and raw sequencing reads are available under the accession numbers PRJNA1128679, SAMN42077069, and SRX27502923, respectively.
